# Impact of stress and stress mindset on prevalence of cardiovascular disease risk factors among first responders

**DOI:** 10.1186/s12889-023-16819-w

**Published:** 2023-10-05

**Authors:** Brian Hendricks, Tyler D. Quinn, Bradley S. Price, Timothy Dotson, Elizabeth A. Claydon, Rodney Miller

**Affiliations:** 1 Department of Epidemiology and Biostatistics, West Virginia University, Morgantown, WV 26506 USA; 2 West Virginia Clinical and Translational Sciences Institute, Morgantown, WV 26506 USA; 3 Department of Management Information Systems, West Virginia University, Morgantown, WV 26506 USA; 4 Department of Social and Behavioral Sciences, West Virginia University, Morgantown, WV 26506 USA; 5 West Virginia Sheriff’s Association, Charleston, WV 25311 USA

**Keywords:** First Responders, Stress mindset, Cardiovascular Disease, Rural

## Abstract

**Background:**

Psychological stress is recognized as an important modifiable risk factor for cardiovascular disease (CVD). Despite its potential significance, few to no studies have evaluated the association between stress, stress mindset, and CVD risk factors among rural first responders. The objectives of this study were to identify relationships between general stress, stress mindset, and CVD risk factors.

**Methods:**

The study sample (n = 148) included those 18 years or older and who currently serve as a first responder, defined as either EMS, firefighter, or law enforcement. Questionnaires captured information on demographics, years of work experience as a first responder, multiple first responder occupations, general stress, stress mindset, and self-reported CVD risk factors. Data were analyzed using regression analyses.

**Results:**

Findings suggest that first responders with a stress-is-negative mindset have significantly higher general stress levels (β = 2.20, p = 0.01). Of note, general stress was not a significant predictor of CVD risk factors (AOR = 1.00, 95%CI = 0.93, 1.08) included in our study. However, a negative stress mindset was statistically significant predictor of CVD risk factors (AOR = 2.82, 95%CI = 1.29, 6.41), after adjusting for general stress and other potential confounders.

**Conclusions:**

Findings suggest that stress mindset is an independent predictor of stress and CVD risk factors among rural first responders. These results have the potential to inform educational and organization level interventions targeting stress appraisal for this vulnerable sub population of workers.

**Supplementary Information:**

The online version contains supplementary material available at 10.1186/s12889-023-16819-w.

## Introduction

First responders (emergency medical services [EMS] workers, firefighters, and law enforcement) are on the front lines of healthcare, public order, and disaster response in America. Despite significant improvements in prevention strategies and understanding over the past 10 years, cardiovascular disease (CVD) remains the most prevalent cause of on-duty deaths among firefighters [1, 2]. Additionally, sudden cardiac events are estimated to account for 7–22% of deaths among law enforcement officers [3], 17% among wildland firefighters [4], and 11% among emergency medical services workers [5]. Reflective of the ongoing national burden of CVD among this worker population, the National Occupational Research Agenda from CDC National Institute for Occupational Safety and Health continues to cite reducing CVD among first responders as a national research priority [6].

The disproportionate burden of CVD among first responders can be related to exposure to many physical and psychological stressors unique to the occupation. For example, repeated exposure to high psychological stress stemming from traumatic events, high workload, high responsibility, and little psychological recovery are well-known exposures related to CVD [7, 8] which is highly prevalent among first responders. Given the nature of their work, they are more likely to have repeated exposures to physical injury, mental health crises, and unpredictable work or sleep schedules [9, 10]. Vulnerabilities of this sub-population are recognized by health agencies, such as CDC and Substance Abuse and Mental Health Services Administration (SAMHSA), who provide national resources on general prevention and management of stress for first responders [11, 12]. Still, first responder populations often suffer in silence given negative perceptions around mental health treatment-seeking [13]. A 2020 systematic review conducted by Spritzer 2020 evaluated 48 articles over ten years of published research. Findings suggested that first responders are at an increased risk of mental health conditions [13]. Unfortunately, this issue is potentially exacerbated within individuals based on sex at birth and different racial and ethnic backgrounds who might experience additional social pressures or discrimination [14]. To address these concerns, many federal agencies supporting first responder populations, such as the U.S. Department of Justice, have called for research which addresses disadvantages or disparities across the intersectionality of sex at birth and race/ethnicity [15].

The concept of psychological stress is recognized as an important modifiable risk factor for CVD (16, 17). This stress response can be triggered when the demand on individuals exceeds their psychosocial bandwidth, or ability to adapt or cope with adversity (18). Demands can take on many forms, such as acute stressors (e.g., death of relatives) or chronic stressors (e.g., work-specific stress) (18). The impact of chronic stressors, including work-related stress, has been linked to an increase in recurrent adverse cardiac events among men and women in previous research (16, 19–21). The adverse effects of stress on cardiovascular health may be modified by coping strategies informed by cognitive appraisal and an individual’s stress mindset [22, 23]. Separate from its direct effects on health, chronic stress also facilitates indirect pathways to CVD. For example, chronic stress can facilitate a variety of unhealthy eating habits associated with CVD risk factors [24].

Previous studies have found that a stress-is-positive mindset is associated with reduced demands on psychosocial resources, such as emotional expression, emotional support seeking, and problem solving [22]. As such the objectives of this study were to evaluate the effects of stress and stress mindset on risk factors for cardiovascular disease among first responders across a range of work responsibilities, years of experience, and demographics. This research places specific emphasis on understanding (1) how stress mindset is related to stress adjusting for years of experience, multiple job responsibilities and demographics, and (2) whether stress mindset is a separate risk factor, aside from general stress, for self-reported CVD risk factors. Study findings are reported in accordance with Strengthening the Reporting of Observational studies in Epidemiology (STROBE) cross-sectional study, as well as the additional reporting guidelines for reporting practices for survey research [25, 26]. Results address a critical need for rigorous studies performed across first responder occupations, within sex at birth categories, and among rural and underserved states. Findings address a gap in the literature and could inform future interventions to more proactively address stress/stress mindset associated with cardiovascular disease among first responders.

## Methods

### Questionnaire design

Demographic questions related to age, race/ethnicity, sex at birth, and approximate household income were guided by U.S. Census Bureau American Community Survey groupings [27]. Marital status, family history of public service, current first responder position, years of first responder experience, paid versus volunteer, currently smoke or chew tobacco were all developed internally. Marital status categories were (1) Married, (2) Widowed, (3) Divorced, and (4) Not Married. Family history of public service was binary (Yes/No). Current occupation categories were (1) EMT, (2) Paramedic, (3) Firefighter, (4) Firefighter/EMT, (5) Firefighter/Paramedic, (6) Uniformed Police Officer, (7) Non-Uniformed Police Office, (8) Investigator, (9) Uniformed Police Officer/Investigator, (10) Non-Uniformed Police Officer/Investigator, and 11) Other; where participants could specify other as free text. Paid versus volunteer position was a binary variable (Paid/Not Paid). Years of experience categories were (1) Less than 1 year, (2) 1 to 2 years, (3) 3 to 4 years, (4) 5 to 7 years, (5) 8 to 9 years, 6)10 to 11 years, 7) 12 to 14 years, and 8) 15 + years. Currently chew or smoke tobacco was binary (Yes/No).

Presence or absence of any CVD risk factor were based upon Yes/No participant responses to whether they had ever been diagnosed with (1) hypertension (high blood pressure), (2) diabetes, (3) chronic kidney disease, (4) chronic obstructive pulmonary disease (COPD), (5) stroke, (6) hyperlipidemia (high-cholesterol). Presence of a negative stress mindset was captured using the “the effects of stress are negative” question from the Stress Mindset-General instrument validated in previous research [28]. Participants indicating “strong agreement” or “agreement” with “the effects of stress are negative” statement was identified as possessing a negative stress mindset. Previous studies found that the General Stress Mindset questionnaire had a high internal consistency of 0.86 (Cronbach’s alpha) [28]. Briefly, stress mindset is a measure of whether individuals believe that general stress is positive or negative on productivity and well-being [28]. Overall stress within the past week was assessed using the Stress Scale questions from the Depression Anxiety Stress Scale-21 (DASS21) questionnaire [29]. Internal consistency of the previously validated DASS-21 Stress Scale was 0.90 [29]. A copy of the questionnaire instrument is included as *Supplemental Files*.

### Study sample

Individuals over 18 years of age who currently serve as a first responder, defined as either EMS, firefighter, or law enforcement were included in this study. The restriction on individuals across the lifespan was due to limitations associated with the age at which residents can apply for any of the first responder positions listed above. Stress exposures, stress responses, and chronic disease risk factors among junior firefighters and paramedics are likely different than their adult counterparts. The electronic survey was created using ESRI Survey 123 software (30), and data were stored securely on a HIPAA compliant ESRI ArcGIS Enterprise (31) server managed by the West Virginia Clinical and Translational Sciences Institute. Recruitment was conducted from October 31, 2022 to November 15, 2022. Recruitment and survey response collection was done through electronic dissemination through internal list servs for the West Virginia Sheriffs Association (which includes Sherriff’s as well as other local law enforcement), Professional Firefighters of West Virginia Association, and WV Healthnet (includes ground and air EMS transport services). Participants received a 25.00-dollar incentive for completing the survey. The survey instrument was to be completed in one sitting and was estimated to take roughly 15 min to complete. This study was approved by the West Virginia Internal Review Board (protocol # 2210658995).

### Statistical analysis

Summary statistics were calculated to visualize mean stress scores and row percentages for demographics and clinical factors for those with stronger stress-is-enhancing mindsets versus those that did not. The DASS-21 Stress Scale for each participant was centered by subtracting the mean from each person’s observed value. This was done to normalize DASS-21 Stress Scales prior to regression. Current positions were dichotomized to identify individuals with multiple first responder roles versus those that did not. This is consistent with previous research that suggests that individuals serving in multiple first responder capacities are at higher risk of a mental health condition [32]. Dichotomizing first responder occupation to multi-job or not, also provides an opportunity to examine multiple functions in the first responder community as an exposure. This is responsive to a need for rigorous research to address first responder needs across the spectrum of occupations [13]. The questionnaire was administered to 156 first responders. This was the limit of funding available for incentives.

Separate linear and logistic regression analyses were conducted to examine the associations between stress, stress mindset, and CVD risk factors included in the study. Variable selection was conducted by calculating chi-square statistics to investigate multicollinearity in categorical predictor variables prior to regression analysis. All statistical analyses were conducted in R [33].

## Results

Among the 156 first responders who participated, 8 (5%) were excluded to perform a complete case analysis. A comprehensive table of summary statistics is provided in Table 1. The mean centered DASS-21 Stress Scale value was 0.59 (std dev = 4.84). Prior to centering the mean for DASS-21 Stress Scale was 8.39 (std dev = 4.84). Row and column percentages are provided for each categorical variable grouped by presence or absence of a negative stress mindset. Row percentages are presented first, directly to the right of the counts. Of the 148 participants with complete data, 76% were male, 65% were married, 36% had an average household income of between 100,000 and 149,999 dollars, 57% have 12 or more years of experience as a first responder, 68% had only one first responder occupation, and 75% did not smoke or chew tobacco. Additionally, 50% of first responders reported having at least one of the CVD risk factors included in this study. More specifically, 37% reported hypertension, 2% reported COPD, 12% reported Diabetes, and 15% reported Hyperlipidemia. Of particular interest, first responders self-reporting a negative stress mindset also reported lower average household incomes, higher percentage of persons not married, and higher prevalence of hypertension, diabetes, and COPD, and presence of any of the CVD risk factors. Chronic kidney disease and stroke are not included in Table 1, as only one individual self-reported experiencing these two conditions. This same individual reported multiple other conditions and are included in the breakdown presented in Table 1.

**Table 1:** Population Description Grouped by Presence or Absence of a Negative Stress Mindset

	Negative Stress Mindset		
**Variable**	**Absent**	**Present**	**Overall**
**Experience**			
Count (%)	78 (52.70%)	70 (47.30%)	148
(Row %)(Col %)			
<= 2 years	4 (57.14%) (5.13%)	3 (42.86%) (4.29%)	7 (100.00%) (4.73%)
3 to 7 years	23 (60.53%) (29.49%)	15 (39.47%) (21.43%)	38 (100.00%) (25.68%)
8 to 11 years	6 (31.58%) (7.69%)	13 (68.42%) (18.57%)	19 (100.00%) (12.84%)
>= 12 years	45 (53.57%) (57.69%)	39 (46.43%) (55.71%)	84 (100.00%) (56.76%)
**Avg Household Income**			
Count (%)	78 (52.70%)	70 (47.30%)	148
(Row %)(Col %)			
<= 49,999 dollars	11 (44.00%) (14.10%)	14 (56.00%) (20.00%)	25 (100.00%) (16.89%)
50,000 to 74,999	19 (54.29%) (24.36%)	16 (45.71%) (22.86%)	35 (100.00%) (23.65%)
75,000 to 99,999	17 (47.22%) (21.79%)	19 (52.78%) (27.14%)	36 (100.00%) (24.32%)
100,000 to 149,999	31 (59.62%) (39.74%)	21 (40.38%) (30.00%)	52 (100.00%) (35.14%)
**Sex**			
Count (%)	78 (52.70%)	70 (47.30%)	148
(Row %)(Col %)			
Male	57 (50.44%) (73.08%)	56 (49.56%) (80.00%)	113 (100.00%) (76.35%)
Female	21 (60.00%) (26.92%)	14 (40.00%) (20.00%)	35 (100.00%) (23.65%)
**Marital Status**			
Count (%)	78 (52.70%)	70 (47.30%)	148
(Row %)(Col %)			
Not Married	12 (42.86%) (15.38%)	16 (57.14%) (22.86%)	28 (100.00%) (18.92%)
Married	53 (55.21%) (67.95%)	43 (44.79%) (61.43%)	96 (100.00%) (64.86%)
Divorced	13 (54.17%) (16.67%)	11 (45.83%) (15.71%)	24 (100.00%) (16.22%)
**Multiple Occupations**			
Count (%)	78 (52.70%)	70 (47.30%)	148
(Row %)(Col %)			
No	53 (51.96%) (67.95%)	49 (48.04%) (70.00%)	102 (100.00%) (68.92%)
Yes	25 (54.35%) (32.05%)	21 (45.65%) (30.00%)	46 (100.00%) (31.08%)
**Tobacco Use**			
Count (%)	78 (52.70%)	70 (47.30%)	148
(Row %)(Col %)			
No	58 (51.79%) (74.36%)	54 (48.21%) (77.14%)	112 (100.00%) (75.68%)
Yes	20 (55.56%) (25.64%)	16 (44.44%) (22.86%)	36 (100.00%) (24.32%)
**Hypertension**			
Count (%)	78 (52.70%)	70 (47.30%)	148
(Row %)(Col %)			
Absent	53 (57.61%) (67.95%)	39 (42.39%) (55.71%)	92 (100.00%) (62.16%)
Present	25 (44.64%) (32.05%)	31 (55.36%) (44.29%)	56 (100.00%) (37.84%)
**Diabetes**			
Count (%)	78 (52.70%)	70 (47.30%)	148
(Row %)(Col %)			
Absent	70 (54.26%) (89.74%)	59 (45.74%) (84.29%)	129 (100.00%) (87.16%)
Present	8 (42.11%) (10.26%)	11 (57.89%) (15.71%)	19 (100.00%) (12.84%)
**COPD**			
Count (%)	78 (52.70%)	70 (47.30%)	148
(Row %)(Col %)			
Absent	78 (53.79%) (100.00%)	67 (46.21%) (95.71%)	145 (100.00%) (97.97%)
Present	0 (0.00%) (0.00%)	3 (100.00%) (4.29%)	3 (100.00%) (2.03%)
**Hyperlipidemia**			
Count (%)	78 (52.70%)	70 (47.30%)	148
(Row %)(Col %)			
Absent	66 (52.38%) (84.62%)	60 (47.62%) (85.71%)	126 (100.00%) (85.14%)
Present	12 (54.55%) (15.38%)	10 (45.45%) (14.29%)	22 (100.00%) (14.86%)
**Any CVD Risk Factor**			
Count (%)	78 (52.70%)	70 (47.30%)	148
(Row %)(Col %)			
Absent	45 (60.81%) (57.69%)	29 (39.19%) (41.43%)	74 (100.00%) (50.00%)
Present	33 (44.59%) (42.31%)	41 (55.41%) (58.57%)	74 (100.00%) (50.00%)

Regression model results are displayed in Tables 2, 3 and 4. Table 2 evaluates whether a negative stress mindset is associated with general stress (measured through DASS-21 Stress Scale), adjusting for potential confounders. Findings suggest that there is a statistically significant association between presence of a negative stress mindset and the general stress levels for participating first responders (β = 2.20, p = 0.01). No other predictors (e.g., years of experience. multiple first responder occupations, or tobacco use) displayed statistically significant associations. Table 3 evaluates whether the odds of previous diagnosis for any of the study CVD risk factors are associated with general stress levels for participating first responders, adjusting for potential confounders. None of the predictors were statistically associated with the odds of a participating first responder self-reporting having a history of any of the study CVD risk factors, including general stress levels (AOR = 1.00, 95%CI = 0.93, 1.08). Lastly, Table 4 evaluates whether a negative stress mindset is an independent risk factor from general stress for any of the CVD risk factors included in our study. Here, general stress levels remained non-significant (AOR = 0.98, 95%CI = 0.91, 1.06) while negative stress mindset was statistically associated with higher prevalence of CVD risk factors in participating first responders (AOR = 2.82, 95%CI = 1.29, 6.41). Regression analyses did not include income, sex, or marital status as these predictors were co-linear with years of experience in bivariate chi-square statistics.

**Table 2:** Ordinary Least Squares Regression to identify adjusted association between general stress scale (outcome) and negative stress mindset. (95% CI = 95% Confidence Interval)

Variable	OLS Coefficient	95% CI	*P*-Value
**Experience**			
<= 2 years	Ref	Ref	Ref
3 to 7 years	1.42	-2.50, 5.35	0.47
8 to 11 years	0.24	-3.98, 4.48	0.91
>= 12 years	1.04	-2.74, 4.82	0.58
**Negative Stress Mindset**			
Absent	Ref	Ref	Ref
Present	2.20	0.64, 3.76	0.01
**Multiple Occupations**			
No	Ref	Ref	Ref
Yes	0.39	-1.28, 2.06	0.64
**Tobacco Use**			
No	Ref	Ref	Ref
Yes	1.52	-0.27, 3.32	0.09

**Table 3:** Logistic regression model to identify adjusted association between odds of past diagnosis with a study CVD risk factor (outcome) and general stress scale. (95% CI = 95% Confidence Interval)

Variable	Adjusted Odds Ratio	95% CI	*P*-Value
**Experience**			
<= 2 years	Ref	Ref	Ref
3 to 7 years	0.43	0.07, 2.60	0.34
8 to 11 years	0.23	0.02, 1.68	0.14
>= 12 years	2.71	0.54, 15.1	0.22
**Multiple Occupations**			
No	Ref	Ref	Ref
Yes	1.11	0.50, 2.46	0.80
**Tobacco Use**			
No	Ref	Ref	Ref
Yes	1.22	0.52, 2.94	0.65
**General Stress Scale**	1.00	0.93, 1.08	0.92

**Table 4:** Logistic regression model to identify adjusted association between odds of past diagnosis with a study CVD risk factor (outcome) and negative stress mindset. (95% CI = 95% Confidence Interval)

Variable	Adjusted Odds Ratio	95% CI	*P*-Value
**Experience**			
<= 2 years	Ref	Ref	Ref
3 to 7 years	0.44	0.07, 2.75	0.36
8 to 11 years	0.16	0.02, 1.28	0.08
>= 12 years	2.79	0.53, 16.5	0.23
**Multiple Occupations**			
No	Ref	Ref	Ref
Yes	1.15	0.51, 2.60	0.74
**Tobacco Use**			
No	Ref	Ref	Ref
Yes	1.34	0.56, 3.27	0.51
**General Stress Scale**	0.98	0.91, 1.06	0.62
**Negative Stress Mindset**			
Absent	Ref	Ref	Ref
Present	2.82	1.29, 6.41	0.01

## Discussion

Stress research and related behavioral health interventions have been applied within public health for over 30 years (34). Many programs and questionnaires designed to capture and treat stress perpetuate notions that stress is negative and should be avoided (34–36). Unfortunately, not all individuals are able to avoid stress. This statement is particularly true for most people during the last three years of the COVID-19 pandemic (37). The disproportionate inability to avoid stress is of considerable public health concern, as these disparities may lead to limited generalizability in effectiveness of stress intervention programs or higher risk of disease among people, places, and time. The concept of a stress mindset is relatively new, and is not commonly applied within stress reappraisal trainings (35). Past research has demonstrated clear associations between a stress-is-positive mindset and the body’s cortisol reactivity and emotional expression to stress [22, 28]. As such, a stress-is-positive mindset approach may be paramount in trainings seeking to attenuate the effects of stress response on health in individuals with unavoidable acute stressors, such as first responders.

To our knowledge this is the first study to report the effects of negative stress mindset on general stress and prevalence of CVD risk factors among first responders in a largely rural and Appalachian state. Study findings indicated that a negative stress mindset was positively associated with increased stress among respondents. Furthermore, a negative stress mindset was associated with a statistically greater prevalence of CVD risk factors in this study. Importantly, our study did not find a statistically significant relationship between general stress and presence of CVD risk factors. This finding is supported by previous research which also found that stress mindset is a distinct and meaningful factor apart from general stress [28]. Interestingly, first responders self-reporting a negative stress mindset also reported lower average household incomes, higher percentage of persons not married, and higher prevalence of hypertension, diabetes, and COPD, and presence of any of the CVD risk factors. This could indicate a pre-disposition to a negative stress mindset for first responders working in communities with poorer social determinants of health. This was not formally evaluated in this study.

Limitations to our approach exist. First, paid versus volunteer status was not included in the analysis despite it being captured in the questionnaire. Among the 148 participants in the sample analyzed, 3 (2%) were volunteers. Lack of representation of volunteer first responders in the sample stem from two likely sources (1) in WV, only fire departments accept volunteers, and (2) firefighter recruitment was done through the Professional Firefighters of West Virginia Association. Most of the members were career firefighters, and unlikely to be serving in a volunteer capacity. Aside from limited generalizability to volunteer firefighters, our study also had limited to no capacity to address how stress mindsets have changed in this population over time or space. This is an important limitation to the cross-sectional design, as the ecology of the stress landscape maybe different within communities and at discreet time points (e.g., COVID-19 pandemic) [37]. Importantly, this limitation is of minimal concern in our study given all questionnaire responses were collected within a short two-month period.

Despite these limitations, this study leveraged an active first responders research network developed through the NIH National Institutes on Minority Health and Health Disparities (NIMHD) Rapid Acceleration of Diagnostics for Underserved Populations (RADx-UP) initiative in West Virginia. This powerful resource enabled rapid dissemination of the questionnaire and recruitment (all 156 completed within two weeks) through first responder networks. Additionally, senior personnel (fire department chiefs, EMS directors, and WV sheriffs Association) all had opportunities to contribute to wording of questions. This presented a novel opportunity to address internal validity of the questionnaire to ensure meaningful interpretation of the results for WV first responders. The analyses addressed a critical need to assess outcomes across individual and combinations first responder occupations, and examined exposure through years of first responder experience. Importantly, these findings highlight clear relationships between stress, stress mindset, and CVD risk factors among rural first responders. Yet, further research is needed to understand how first responders experience and describe stress and how these experiences influence mechanistic pathways related to cardiovascular disease. Importantly, this study utilized stress mindset, which is only part of the cognitive appraisal process described by Lazarus and Folkman 1984 [23]. More information is needed to holistically evaluate cognitive appraisal process and whether other indicators of primary and secondary appraisal are more informative to stress interventions among rural first responder populations.

### Electronic supplementary material

Below is the link to the electronic supplementary material.

Supplementary Material 1

## Data Availability

The datasets generated and/or analyzed during the current study are not publicly available, as they were primary data collection as part of a NIH funded project. However, they may be available from the corresponding author on reasonable request.

## References

[1] Farioli A, Yang J, Teehan D, Baur DM, Smith DL, Kales SN (2014). Duty-related risk of sudden cardiac death among young US firefighters. Occup Med (Chic Ill).

[2] United States Fire Administration. Firefighter Fatality Retrospective Study [Internet]. 2002 [cited 2023 Aug 9]. Available from: https://www.Users/baw00017/Downloads/fa-220.pdf.

[3] Zimmerman FH (2012). Cardiovascular Disease and Risk factors in Law Enforcement Personnel. Cardiol Rev.

[4] Butler C, Marsh S, Domitrovich JW, Helmkamp J (2017). Wildland firefighter deaths in the United States: a comparison of existing surveillance systems. J Occup Environ Hyg.

[5] Maguire BJ, Hunting KL, Smith GS, Levick NR (2002). Occupational fatalities in emergency medical services: a hidden crisis. Ann Emerg Med.

[6] NORA Public Safety Council. National Occupational Research Agenda for Public Safety. National Institute for Occupational Safety and Health; 2019.

[7] Dar T, Radfar A, Abohashem S, Pitman RK, Tawakol A, Osborne MT (2019). Psychosocial stress and cardiovascular disease. Curr Treat Options Cardiovasc Med.

[8] Angleman AJ, Van Hasselt VB, Schuhmann BB (2022). Relationship between posttraumatic stress symptoms and Cardiovascular Disease Risk in Firefighters. Behav Modif.

[9] SAMHSA, First Responders. Behavioral Health Concerns, Emergency Response, and Trauma [Internet]. 2018 [cited 2023 Aug 9]. Available from: https://www.samhsa.gov/sites/default/files/dtac/supplementalresearchbulletin-firstresponders-may2018.pdf.

[10] Stanley IH, Boffa JW, Hom MA, Kimbrel NA, Joiner TE (2017). Differences in psychiatric symptoms and barriers to mental health care between volunteer and career firefighters. Psychiatry Res.

[11] SAMHSA. First Responders and Disaster Responders Resource Portal [Internet]. 2023 [cited 2023 Aug 9]. Available from: https://www.samhsa.gov/dtac/disaster-responders.

[12] Centers for Disease Control and Prevention. Emergency Responders: Tips for taking care of yourself [Internet]. 2018 [cited 2023 Aug 9]. Available from: https://emergency.cdc.gov/coping/responders.asp.

[13] Spitzer A. First responders and PTSD: a literature review. J Emerg Med Serv. 2020.

[14] Jahnke SA, Poston WC, Haddock CK, Jitnarin N, Hyder ML, Horvath C (2012). The health of women in the US fire service. BMC Womens Health.

[15] U.S. Department of Justice. Women in Policing: Breaking Barriers and Blazing a Path [Internet]. [cited 2022 Nov 15]. Available from: https://www.ojp.gov/pdffiles1/nij/252963.pdf.

[16] Hjemdahl P, Rosengren A, Steptoe A (2012). Stress and Cardiovascular Disease.

[17] Osborne MT, Shin LM, Mehta NN, Pitman RK, Fayad ZA, Tawakol A (2020). Disentangling the links between psychosocial stress and cardiovascular disease. Circ Cardiovasc Imaging.

[18] Steptoe A, Kivimäki M (2012). Stress and cardiovascular disease. Nat Rev Cardiol.

[19] Aboa-Éboulé C, Brisson C, Maunsell E, Mâsse B, Bourbonnais R, Vézina M (2007). Job strain and risk of acute recurrent coronary heart disease events. JAMA.

[20] Aboa-Éboulé C, Brisson C, Maunsell E, Bourbonnais R, Vézina M, Milot A (2011). Effort-reward imbalance at work and recurrent coronary heart disease events: a 4-year prospective study of post-myocardial infarction patients. Psychosom Med.

[21] Laszlo KD, Ahnve S, Hallqvist J, Ahlbom A, Janszky I (2010). Job strain predicts recurrent events after a first acute myocardial infarction: the Stockholm Heart Epidemiology Program. J Intern Med.

[22] Horiuchi S, Tsuda A, Aoki S, Yoneda K, Sawaguchi Y. Coping as a mediator of the relationship between stress mindset and psychological stress response: a pilot study. Psychol Res Behav Manag. 2018;47–54. 10.2147/PRBM.S150400 PMC5840190 29535562

[23] Lazarus R, Folkman S (1984). Stress, Appraisal and Coping.

[24] Morera LP, Marchiori GN, Medrano LA, Defagó MD, Stress. Dietary patterns and Cardiovascular Disease: a Mini-Review. Front Neurosci. 2019;13. 10.3389/fnins.2019.01226 PMC6861179 31780892

[25] STROBE. Cross-Sectional Studies Checklist [Internet]. [cited 2022 Oct 15]. Available from: https://www.strobe-statement.org/.

[26] Bennett C, Khangura S, Brehaut JC, Graham ID, Moher D, Potter BK (2011). Reporting guidelines for survey research: an analysis of published guidance and reporting practices. PLoS Med.

[27] U.S. Census Bureau. Advanced Search Tools [Internet]. [cited 2022 Oct 9]. Available from: https://data.census.gov/cedsci/advanced.

[28] Crum AJ, Salovey P, Achor S (2013). Rethinking stress: the role of mindsets in determining the stress response. J Pers Soc Psychol.

[29] Henry JD, Crawford JR (2005). The short-form version of the Depression anxiety stress scales (DASS‐21): construct validity and normative data in a large non‐clinical sample. Br J Clin Psychol.

[30] ESRI. Survey123 [Internet]. [cited 2022 Oct 9]. Available from: https://www.esri.com/en-us/arcgis/products/arcgis-survey123/overview.

[31] ESRI. ArcGIS Enterprise and Portal [Internet]. [cited 2022 Oct 9]. Available from: https://enterprise.arcgis.com/en/portal/.

[32] Stanley IH, Hom MA, Joiner TE (2016). A systematic review of suicidal thoughts and behaviors among police officers, firefighters, EMTs, and paramedics. Clin Psychol Rev.

[33] Core Team R, Team A. RC. R: A language and environment for statistical computing. R Foundation for Statistical Computing, Vienna, Austria. 2012. 2022.

[34] Crum A. Evaluating a mindset training program to unleash the enhancing nature of stress. In: Academy of Management Proceedings. Academy of Management Briarcliff Manor, NY 10510; 2011. p. 1–6.

[35] Jamieson JP, Crum AJ, Goyer JP, Marotta ME, Akinola M (2018). Optimizing stress responses with reappraisal and mindset interventions: an integrated model. Anxiety Stress Coping.

[36] Cohen S, Kamarck T, Mermelstein R (1983). A Global measure of perceived stress. J Health Soc Behav.

[37] Van Bavel JJ, Baicker K, Boggio PS, Capraro V, Cichocka A, Cikara M (2020). Using social and behavioural science to support COVID-19 pandemic response. Nat Hum Behav.

