# Supplementary material for: Impact of stress and stress mindset on prevalence of cardiovascular disease risk factors among first responders
Source: BMC Public Health. 2023 Oct 5;23:1929. doi: 10.1186/s12889-023-16819-w (PMC10557332; doi:10.1186/s12889-023-16819-w)
Supplement: Supplementary file 1 — Supplementary Material 1 [file 12889_2023_16819_MOESM1_ESM.pdf]

First Responder Stress and Cardiovascular Health Survey

Dear Participant,
Thank you for your interest in this research. The purpose of this quick 38-item survey is to better understand the impact of stress on cardiovascular health for first responders. A secondary goal is to determine whether the effects of stress on cardiovascular health change based upon how important being a first responder is to your personal identity (e.g., centrality).

Participants can skip any question they choose not to answer, except for the first question asking for your consent to collect your survey responses. This study was approved by the WVU IRB (Protocol # 2210658995).

The first 150 first responders to complete this survey will receive a 25-dollar gift card as a thank you for their time and consideration!
** To receive this incentive, please enter your phone or email in the space provided at the end of the survey. Our study staff will reach out to collect your mailing information.

Sincerely,
Dr. Brian Hendricks
Assistant Professor of Epidemiology
West Virginia University School of Public Health
Email: bmhendricks@hsc.wvu.edu

Informed Consent

I agree to provide my most sincere answers to the questions below, and understand that research using my information will be de-identified to protect my identity

- ○ Yes
- ○ No

Demographics Section

Age Group

- ○ 19 years of age or less
- ○ 20 to 29
- ○ 30 to 39
- ○ 40 to 49
- ○ 50 to 59
- ○ 60+

Sex at Birth

- ○ Male
- ○ Female

Race

- White
- Black
- Asian
- American Indian / Alaskan Native
- Native Hawaiian/Other Pacific Islander
- Mixed Race

Family History of First Responder Service

- Yes
- No

Marital Status

- Not Married
- Married
- Widowed
- Divorced

Approximate Household Income

- 24,999 dollars or less
- 25,000 to 49,999
- 50,000 to 74,999
- 75,000 to 99,999
- 100,000 to 149,999

Current Position

- EMT
- Paramedic
- Firefighter
- Firefighter/EMT
- Firefighter/Paramedic
- Uniformed Police Officer
- Non-Uniformed Police Officer
- Investigator
- Uniformed Police Officer/Investigator
- Non-Uniformed Police Officer/Investigator

In your current position are you career (paid) or volunteer (unpaid)

- Paid

- ○ Volunteer

How many years have you held a position as a first responder?

- ○ Less than 1 year
- ○ 1 to 2 years
- ○ 3 to 4 years
- ○ 5 to 7 years
- ○ 8 to 9 years
- ○ 10 to 11 years
- ○ 12 to 14 years
- ○ 15+ years

Physical Health Section

Have you ever been diagnosed by a doctor with any of the following?

- ○ Hypertension (High Blood Pressure)
- ○ Diabetes
- ○ Chronic Kidney Disease
- ○ COPD
- ○ Peripheral Artery Disease
- ○ Coronary Heart Disease
- ○ Stroke
- ○ Hyperlipidemia (High Cholesterol)
- ○ Cancer
- ○ None

Do you currently smoke or chew tobacco?

- ○ Yes
- ○ No

Stress Section

At times these questions may seem repetitive. Please answer each as they are used to compute an index of stress.

In the past week have you experienced any of the following?

I find it hard to wind down

○	○	○	○
Did not apply to me at all	Applied to me to some degree, or some of the time	Applied to me to a considerable degree, or a good part of time	Applied to me very much, or most of the time

I tend to over-react to situations

Did not apply to me at all	Applied to me to some degree, or some of the time	Applied to me to a considerable degree, or a good part of time	Applied to me very much, or most of the time

I feel that I have a lot of nervous energy

Did not apply to me at all	Applied to me to some degree, or some of the time	Applied to me to a considerable degree, or a good part of time	Applied to me very much, or most of the time

I find myself getting agitated

Did not apply to me at all	Applied to me to some degree, or some of the time	Applied to me to a considerable degree, or a good part of time	Applied to me very much, or most of the time

I find it difficult to relax

Did not apply to me at all	Applied to me to some degree, or some of the time	Applied to me to a considerable degree, or a good part of time	Applied to me very much, or most of the time

I am intolerant of anything that keeps me from getting on with what I am doing

Did not apply to me at all	Applied to me to some degree, or some of the time	Applied to me to a considerable degree, or a good part of time	Applied to me very much, or most of the time

I feel that I am rather touchy (e.g., overly sensitive)

Did not apply to me at all	Applied to me to some degree, or some of the time	Applied to me to a considerable degree, or a good part of time	Applied to me very much, or most of the time
◯	◯	◯	

Stress Mindset

At times these questions may seem repetitive. Please answer each as they are used to compute an index of your personal mindset around stress.
Please rate the extent to which you agree or disagree with the following statements.

The effects of stress are negative and should be avoided.

◯ Strongly Disagree ◯ Disagree ◯ Neither Agree nor Disagree ◯ Agree ◯ Strongly Agree

Experiencing stress facilitates my learning and growth.

◯ Strongly Disagree ◯ Disagree ◯ Neither Agree nor Disagree ◯ Agree ◯ Strongly Agree

Experiencing stress depletes my health and vitality.

◯ Strongly Disagree ◯ Disagree ◯ Neither Agree nor Disagree ◯ Agree ◯ Strongly Agree

Experiencing stress enhances my performance and productivity.

◯ Strongly Disagree ◯ Disagree ◯ Neither Agree nor Disagree ◯ Agree ◯ Strongly Agree

Experiencing stress inhibits my learning and growth.

◯ Strongly Disagree ◯ Disagree ◯ Neither Agree nor Disagree ◯ Agree ◯ Strongly Agree

Experiencing stress improves my health and vitality.

◯ Strongly Disagree ◯ Disagree ◯ Neither Agree nor Disagree ◯ Agree ◯ Strongly Agree

Experiencing stress debilitates my performance and productivity.

◯ Strongly Disagree ◯ Disagree ◯ Neither Agree nor Disagree ◯ Agree ◯ Strongly Agree

The effects of stress are positive and should be utilize.

◯ Strongly Disagree ◯ Disagree ◯ Neither Agree nor Disagree ◯ Agree ◯ Strongly Agree

Please enter your phone number or email in the space provided below if you would like to receive the 25 dollar incentive.

____

Thank you so very much for your time and consideration!
